# Global Trends in Avian Pathogenic *Escherichia coli* Research: A Bibliometric Analysis (2000 to 2025)

**DOI:** 10.3390/vetsci13101100

**Published:** 2026-10-16

**Authors:** Mei Xue, Jingjing Liu, Weiqiang Lei, Ping Zhang

**Affiliations:** College of Animal Science and Food Engineering, Jinling Institute of Technology, Nanjing 210038, China

**Keywords:** avian pathogenic *Escherichia coli*, bibliometric analysis, research trends

## Abstract

Avian pathogenic *Escherichia coli* (APEC), a subgroup of extraintestinal pathogenic *E. coli* (ExPEC), causes infections in poultry and may pose a public health risk. Although previous reviews have examined the epidemiology, genotypes, pathogenicity, antimicrobial resistance, and zoonotic risk of APEC, its global research landscape and development trends have not been systematically evaluated. This study analyzed 1121 APEC-related publications published from 2000 to 2025 and indexed in the Web of Science Core Collection (WoSCC). Bibliometrix, VOSviewer, and CiteSpace were used to examine publication trends, countries, institutions, journals, citations, and keywords. The number of publications increased steadily, with more rapid growth after 2019. China produced the most publications, whereas the USA received the most citations. Iowa State University, Yangzhou University, and Anhui Agricultural University were the leading institutions, and *Poultry Science*, *Veterinary Microbiology*, and *Avian Diseases* were the most productive journals. Keyword co-occurrence, clustering, and burst analyses identified infection mechanisms, molecular identification, antimicrobial resistance, and immune responses as the main research topics. These findings provide an overview of the development of APEC research and may support future studies on broad-spectrum vaccines, phage therapy, and microbiota-based interventions for APEC prevention and control.

## Simple Summary

Avian pathogenic *Escherichia coli* (APEC) is an important cause of poultry disease. Although many studies have investigated APEC epidemiology, pathogenicity, antimicrobial resistance, and zoonotic potential, the overall development of APEC research has not been systematically assessed. This study analyzed 1121 publications on APEC published between 2000 and 2025 using bibliometric tools. The results showed a steady increase in publication output, particularly after 2019. China contributed the largest number of publications, while the United States had the greatest citation impact. Major research institutions included Iowa State University, Yangzhou University, and Anhui Agricultural University. The main research topics were infection mechanisms, molecular identification, antimicrobial resistance, and immune responses. These findings summarize the development of APEC research and identify potential directions for future work, including broad-spectrum vaccines, phage therapy, and microbiota-based strategies for disease prevention and control.

## 1. Introduction

The Avian colibacillosis is a major infectious disease that primarily affects broiler chickens and turkeys between 3 and 12 weeks of age. It is characterized by respiratory disease, fibrinous lesions, septicemia, and systemic infection, resulting in substantial reductions in poultry productivity [1,2]. Avian pathogenic *Escherichia coli* (APEC) is the principal causative agent of avian colibacillosis [3]. The virulence repertoire of APEC is complex and encompasses adhesins, toxins, iron acquisition systems, lipopolysaccharide, and invasins. Numerous virulence-associated genes have been identified, many of which are located on pathogenicity islands, plasmids, or other mobile genetic elements [4,5]. In addition, APEC belongs to the extraintestinal pathogenic *E. coli* (ExPEC) group, which also includes uropathogenic *E. coli* (UPEC) and neonatal meningitis-associated *E. coli* (NMEC) [6]. Although APEC has not been directly linked to human infections, accumulating evidence suggests that some strains may have the capacity to cross species barriers [7,8]. For example, APEC O25-ST131 isolates recovered from poultry in Georgia were identified as globally disseminated high-risk clones. These isolates shared genetic features with UPEC strains and survived and proliferated in both chicken serum and human urine, suggesting their potential for zoonotic transmission and human infection [9]. Conversely, evidence suggests that transmission may also occur from humans to poultry. In Korea, an integrated analysis of an rpoB sequence type (RST) network and comparative genomic data indicated that human ExPEC of RST46-1 had been transmitted to poultry and subsequently evolved within poultry populations, raising concerns about the reverse zoonotic transmission of human-derived ExPEC [10].

Antibiotics have long been used for the prevention and treatment of APEC infections. However, the extensive and often inappropriate use of antibiotics has contributed to the emergence and dissemination of multidrug-resistant strains. For example, a study conducted in Bangladesh reported multidrug resistance in all 89 APEC isolates examined [11]. Similarly, Nawaz et al. reported that most APEC isolates collected from four provinces in China were multidrug resistant. Among the antimicrobial agents tested, enrofloxacin had the highest resistance rate, followed by cefepime and amoxicillin–clavulanic acid [12]. In Algeria, APEC isolates recovered from diseased chickens in the Tizi Ouzou region showed high levels of resistance to tetracycline, ampicillin, and ciprofloxacin [13]. In Japan, an O78-ST23 APEC strain producing CTX-M-55-type extended-spectrum β-lactamase (ESBL) and resistant to fluoroquinolones was identified on a farm in Kagoshima. The repeated isolation of this strain from the same farm over a two-year period suggested its persistence and possible circulation within the farm environment [14]. To improve the prevention and control of APEC infections, researchers have investigated vaccines and various alternatives to antibiotics, including probiotics, lytic bacteriophages, and organic acids. Recent studies have assessed an HMT13-adjuvanted inactivated vaccine targeting APEC serogroups O36, O78, and O109, as well as polyvalent inactivated vaccines developed based on the molecular characteristics of APEC isolates from Jiangxi Province, China. Together, these studies support the development of multivalent inactivated vaccines tailored to locally prevalent APEC strains [15,16]. Dietary supplementation with *Enterococcus faecium* 11181 reduced local and systemic inflammation in chickens infected with APEC O78 [17]. *Lactiplantibacillus plantarum* ATS1 alleviated APEC-induced tissue injury and modulated host immune responses [18]. The lytic bacteriophage CABI-SEA 2301 also showed potential for controlling APEC infections [19].

A substantial body of primary research and review literature has examined the detection, epidemiology, pathogenicity, multidrug resistance, and zoonotic potential of APEC, together with the development of alternatives to antibiotics. Bibliometric analysis offers a systematic means of examining the development of a research field, identifying its major themes and emerging trends, and revealing gaps in the literature. Bibliometric studies have examined related fields, including *E. coli* O157, antimicrobial resistance, and poultry diseases [20,21,22]. However, these studies generally focused on specific pathogens, diseases, or research topics. To the best of our knowledge, the global development and thematic evolution of APEC research have not yet been comprehensively assessed. Accordingly, this study aimed to characterize publication trends, major contributors, and research themes in the APEC literature using bibliometric and visualization methods, thereby providing an overview of the field and informing future research.

## 2. Bibliometric Data Collection and Analysis

### 2.1. Data Source and Search Strategy

**Figure 1:**
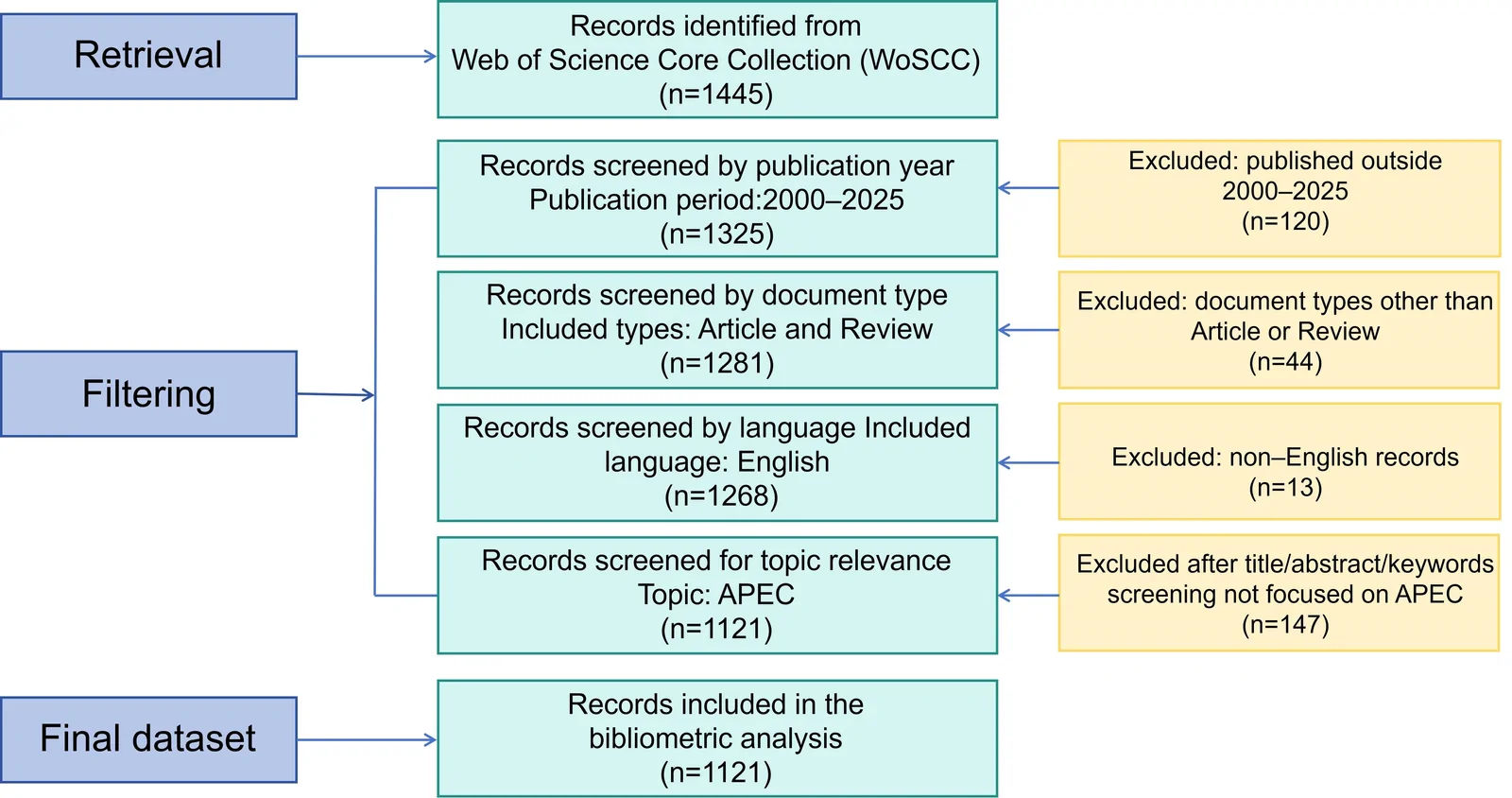
PRISMA flow diagram of the literature identification, screening, eligibility assessment, and inclusion process.

A literature search was conducted in the WoSCC database to identify publications on APEC and avian colibacillosis published from 2000 to 2025. WoSCC provides standardized citation and bibliographic records compatible with widely used bibliometric tools, supporting the reproducible analysis of global scientific output. The following search query was applied: TS = ((“avian pathogenic *Escherichia coli*” OR “avian pathogenic *E. coli*” OR “avian colibacillosis” OR “poultry colibacillosis” OR “avian colisepticemia” OR “avian colisepticaemia”) OR (APEC AND (avian OR poultry OR chicken OR chickens OR broiler OR broilers OR layer OR layers OR turkey OR turkeys OR duck OR ducks OR bird OR birds) AND (“*Escherichia coli*” OR “*E. coli*”)). The search was restricted to publications and reviews published in English. All records were exported as plain-text files with full records and cited references. Then the retrieved records were manually screened by two reviewers independently to ensure the accuracy and relevance of the dataset. Records were included if they focused on APEC, avian colibacillosis, poultry-associated pathogenic *E. coli*, or related topics such as pathogenicity, epidemiology, antimicrobial resistance, diagnosis, prevention, and control. Records were excluded if *E. coli* was mentioned only as a general foodborne or environmental microorganism, if the study focused exclusively on human or non-avian hosts, or if poultry was unrelated to the research topic. Duplicate records and records that did not meet the predefined criteria for publication year, document type, language, or topic relevance were removed. Full texts were reviewed when relevance could not be determined from the title and abstract. Following screening, 1121 eligible publications were included in the final analysis. The resulting dataset was cleaned and imported into bibliometric software for quantitative analysis and visualization. This review was conducted in accordance with the Supplementary Material PRISMA 2020 (Preferred Reporting Items for Systematic Reviews and Meta-Analyses) guidelines. The literature selection process is presented in Figure 1.

### 2.2. Data Analysis and Visualization

A comprehensive bibliometric analysis of APEC research was performed using the R package bibliometrix (version 4.3.0), VOSviewer (version 1.6.19), and CiteSpace (version 6.4.R1). Bibliometrix was used for descriptive analyses of the included publications, including annual publication output and growth trends, contributions by countries or regions and institutions, journal activity, highly cited publications, and citation impact [23]. These analyses provided an overview of the development of APEC research and the global distribution of scholarly contributions. VOSviewer was used to construct and visualize bibliometric networks using network, overlay, and density visualizations, allowing collaboration patterns, clustering structures, and the intensity of research activity to be assessed [24]. Keyword co-occurrence analysis was conducted to identify the major research themes and their relationships within the field. CiteSpace was used to explore the intellectual structure and research frontiers of APEC research. Keyword burst detection was performed to identify topics that received rapidly increasing attention during specific periods and to trace emerging research trends [25]. Co-citation and cluster analyses were also conducted to identify influential references and major components of the field’s knowledge base.

## 3. Bibliometric Results and Thematic Discussion

### 3.1. Annual Publication Output and Growth Trends

**Figure 2:**
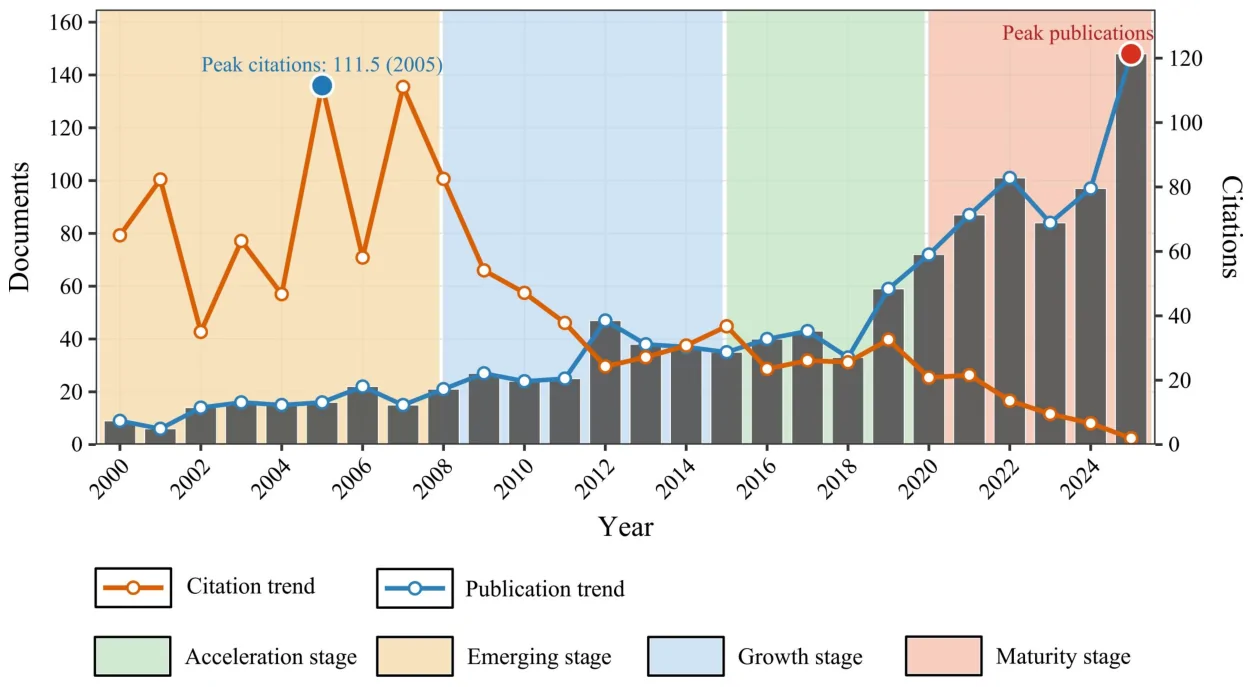
Annual scientific production and citation impact, 2000–2025. Grey bars represent the annual number of publications, while the blue and orange lines show publication and citation trends, respectively. The left and right axes indicate the annual number of documents and the original citation counts, respectively. Shaded background bands identify four developmental stages: the emerging stage (2000–2007), growth stage (2008–2014), acceleration stage (2015–2019), and maturity stage (2020–2025). Publication output peaked in 2025, whereas citation impact reached its highest level in 2005.

Annual publication output and citation trends reflect the temporal development, accumulation of knowledge, and changing scholarly influence of a research field and can help indicate its developmental stage and academic prominence [26]. As shown in Figure 2, the annual number of APEC-related publications generally increased from 2000 to 2025. Between 2000 and 2011, however, annual output remained low and showed only modest year-to-year variation, with fewer than 10 publications in some years and just over 20 in others. This relatively low output suggests that APEC research was still at an early stage of development. The number of publications rose markedly to 47 in 2012 and increased more rapidly after 2019, from 59 in 2019 to 72 in 2020, 87 in 2021, and 101 in 2022. Despite minor fluctuations in 2023 and 2024, annual output remained well above that of earlier years and peaked at 148 publications in 2025. Overall, these findings indicate that APEC research has shifted from gradual growth to rapid expansion, reflecting increasing interest in its relevance to poultry health, pathogenic mechanisms, antimicrobial resistance, and disease prevention and control.

In contrast to the overall increase in publication output, the average number of citations per publication decreased over the study period. Average citation counts were relatively high between 2000 and 2008, reaching 111.50 in 2005 and 111.07 in 2007. These values suggest that some early publications were frequently cited in subsequent research. After 2009, the average declined gradually, with a more marked decrease after 2012 and a sharper decline from 2020 onward. Citation-based indicators are strongly influenced by publication age. Recent articles have had less time to accumulate citations than older publications. Consequently, the apparent decline in average citations in recent years should not be interpreted as evidence of reduced research quality or scientific influence.

### 3.2. Geographical Distribution of Research Output

Analysis of publication patterns by country and region provides insight into the geographical distribution of research output, regional research strengths, and international collaboration within a specific field [27]. From 2000 to 2025, researchers from 80 countries and regions produced 1121 publications on APEC. As shown in Table 1, China was the leading contributor, with 328 publications (29.26% of the total), followed by the USA with 150 publications (13.38%) and Brazil with 89 publications (7.94%). Collectively, these three countries accounted for more than half of the total research output, suggesting a substantial geographical concentration of APEC research. The remaining countries in the top 10 were Canada, Iran, Egypt, France, the UK, South Korea, and Denmark, each contributing less than 4% of the total output.

In terms of citation performance, the USA received the highest total number of citations (7332), averaging 48.88 citations per paper, indicating its substantial citation impact in the field. China ranked second in total citations (4592), but had a lower average citation count of 14.00 per paper. Thus, despite its large publication output, China’s average citation count remained lower than that of the USA. France contributed only 32 papers but recorded the highest average citation count among the 10 most productive countries, at 56.31 citations per paper. Canada and the UK also had high average citation counts, at 48.68 and 47.03 citations per paper, respectively. These findings indicate that several European and North American countries performed strongly in terms of citations per paper. Overall, China led in publication output, whereas the USA had the highest total citation count.

The extent of international collaboration varied considerably across countries. China produced 298 single-country publications and 30 multiple-country publications, corresponding to an MCP ratio of 9.15%. The MCP ratios for the USA, Brazil, Canada, and South Korea were 16.00%, 17.98%, 24.39%, and 21.43%, respectively. Higher ratios were observed for Egypt, Denmark, the UK, and France, at 42.42%, 40.00%, 34.38%, and 34.38%, respectively. Egypt produced 14 multiple-country publications out of 33 records, while Denmark produced 10 multiple-country publications out of 25 records. Although Egypt had a slightly higher level of international collaboration, Denmark showed a higher average citation impact (24.64 citations per publication) than Egypt (19.58). These findings indicate that international collaboration accounted for a larger proportion of publications from Egypt, Denmark, France, and the UK than from most other leading contributors.

**Figure 3:**
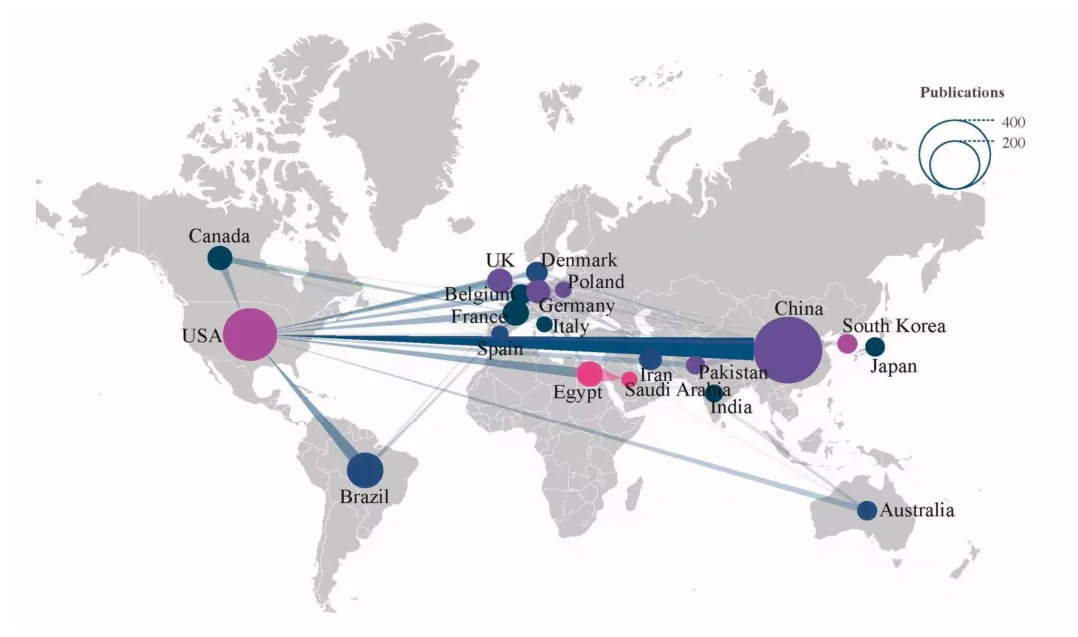
International collaboration among countries in APEC research, 2000–2025. Node size represents the number of publications produced by each country, whereas the thickness of connecting lines reflects the strength of bilateral collaboration.

As shown in Figure 3, China and the United States have the strongest collaborative link and constitute the most active bilateral research partnership in the network. The United States also collaborates extensively with countries in the Americas, Europe, and Oceania. In contrast, China’s international cooperation focuses largely on the United States, while its ties with other countries remain comparatively weak. European countries form a close regional cluster through frequent cross-border collaboration. Overall, the network displays a typical core periphery structure, in which European countries and the United States maintain denser multilateral cooperation networks. Although China has the highest publication output, its international collaboration remains relatively limited in geographical scope. International collaboration may be influenced by poultry production, the burden of avian colibacillosis, research funding, institutional capacity, policy support and other aspects. However, these factors were not directly assessed.

### 3.3. Institutional Productivity and Citation Impact

Analysis of institutional productivity can identify the major research institutions in a field [28]. A total of 1135 institutions contributed to the 1121 publications, and the 10 most productive are listed in Table 2. Iowa State University ranked first with 75 publications (6.69%), followed by Yangzhou University with 69 (6.16%). Anhui Agricultural University and Nanjing Agricultural University tied for third, with 65 publications each (5.80%). The Chinese Academy of Agricultural Sciences and the University of Copenhagen contributed 39 (3.48%) and 30 publications (2.68%), respectively. The National Research Institute for Agriculture, Food and Environment and the University of Georgia each contributed 26 publications (2.32%), while North Dakota State University and the University of Minnesota each contributed 25 (2.23%). Eight of the 10 most productive institutions were located in China or the USA. China was particularly prominent, with three institutions among the four highest-ranked contributors. The remaining two institutions, located in Denmark and France, jointly contributed 56 publications (5.00%).

Publication output and citation impact varied considerably across institutions, suggesting that higher productivity did not necessarily correspond to greater citation impact. Iowa State University ranked first in both publication output and total citations, with 75 publications and 4706 citations, corresponding to an average of 62.75 citations per publication. Despite producing only 25 publications, North Dakota State University received 2952 citations and had the highest average citation count among the institutions analyzed, at 118.08 citations per publication. The University of Minnesota also produced 25 publications, which received 2224 citations, or an average of 62.75 citations per publication. The National Research Institute for Agriculture, Food and Environment received 2060 citations for its 26 publications, corresponding to an average of 79.23 citations per publication. Although Chinese institutions contributed substantially to publication output, their average citation counts were generally lower. The corresponding values for Yangzhou University, Anhui Agricultural University, Nanjing Agricultural University, and the Chinese Academy of Agricultural Sciences were 12.84, 12.91, 18.26, and 21.05 citations per publication, respectively.

### 3.4. Journal Distribution and Source Impact

Journal analysis can help identify the main publication outlets and assess the disciplinary breadth of a research field [29]. In the present study, the journals publishing APEC-related research were examined, and the 10 most productive journals are listed in Table 3. *Poultry Science* was the most productive journal, publishing 101 publications (9.01% of the total output), followed by *Veterinary Microbiology* with 81 publications (7.23%). *Avian Diseases* and *Avian Pathology* ranked third and fourth, with 60 (5.35%) and 53 publications (4.73%), respectively. Several journals with broader or more fundamental scopes, including PLOS ONE, *Microbial Pathogenesis*, *Infection and Immunity*, and *Microorganisms*, were also among the leading publication outlets. Overall, the distribution of publications across these journals suggests that APEC research extends beyond avian medicine to include pathogenic mechanisms, host immune responses, molecular epidemiology, and public health risks, reflecting the interdisciplinary nature of the field.

The 2025 impact factors of the 10 most productive journals ranged from 1.3 to 4.7. Among the journals examined, *Microorganisms* had the highest impact factor, at 4.7, together with a mean citation count of 19.05 per article. *Poultry Science* had the second-highest impact factor, at 4.5, but a lower mean citation count of 14.66 per article. *Veterinary Microbiology* ranked first in total citations, recording 2549 total citations and a mean of 31.47 citations per article. Although *Infection and Immunity* contributed only 28 publications, representing 2.50% of the total output, these articles accumulated 2241 citations, corresponding to a mean of 80.04 citations per article-the highest value among the journals examined. In contrast, *Poultry Science*, despite being the most productive journal, had a relatively low mean of 14.66 citations per article, possibly because of its emphasis on applied research and practical issues in poultry production.

### 3.5. Highly Cited Publications

Highly cited publications often reflect the established knowledge base and emerging research priorities within a field [28]. As shown in Table 4, most of the 10 most frequently cited publications on APEC were published between 2005 and 2008 and focused primarily on APEC identification and its zoonotic potential. Johnson was the first author of four of these publications, whereas Rodriguez-Siek and Ewers each served as first author of two. The most frequently cited publication was a review by Sarowska et al., published in *Gut Pathogens* in 2019, which had received 510 citations. The review summarized the virulence factors, epidemiological distribution, and potential transmission routes of ExPEC from diverse sources, as well as the molecular mechanisms underlying its progression from intestinal colonization to invasive extraintestinal infection [30]. The second most frequently cited publication, published by Ewers et al. in 2007, had received 446 citations. By comparing the phylogenetic relationships and virulence gene profiles of APEC, UPEC, and NMEC isolates, the authors identified substantial genetic overlap among these pathotypes and proposed that certain APEC strains may serve as reservoirs of virulence genes and ColV plasmids associated with human ExPEC [31]. Another highly cited study, published by Johnson et al. in the *Journal of Clinical Microbiology* in 2008, had received 370 citations. The authors selected five genes strongly associated with highly pathogenic APEC strains (*iutA*, *hlyF*, *iss*, *iroN*, and *ompT*) as molecular markers and incorporated them into a multiplex PCR assay. This approach provided a practical method for the rapid identification and assessment of the virulence potential of APEC isolates [5].

Comparative genomic analyses have further characterized the genetic relatedness between APEC and human ExPEC, particularly with respect to their serogroups, phylogenetic backgrounds, virulence-gene profiles, and plasmid carriage patterns [31,32]. Consistent with these observations, the complete genome sequence of APEC O1:K1:H7 exhibits substantial similarity to those of several human ExPEC isolates. Although these genomic similarities support a close genetic relationship, they do not, on their own, demonstrate foodborne transmission of APEC to humans. Further evidence is provided by mixed phylogenetic clusters containing both avian and human isolates, which were predominantly composed of serogroups O1, O2, and O18. Among the isolates within these clusters, 58% belonged to sequence type 95, and more than 90% were classified into phylogenetic group B2. Collectively, these findings suggest that certain APEC subpopulations may possess zoonotic potential and may be capable of causing infection in both avian and human hosts [6,33].

### 3.6. Research Hotspots and Thematic Evolution

#### 3.6.1. Keyword Co-Occurrence Network Analysis

**Figure 4:**
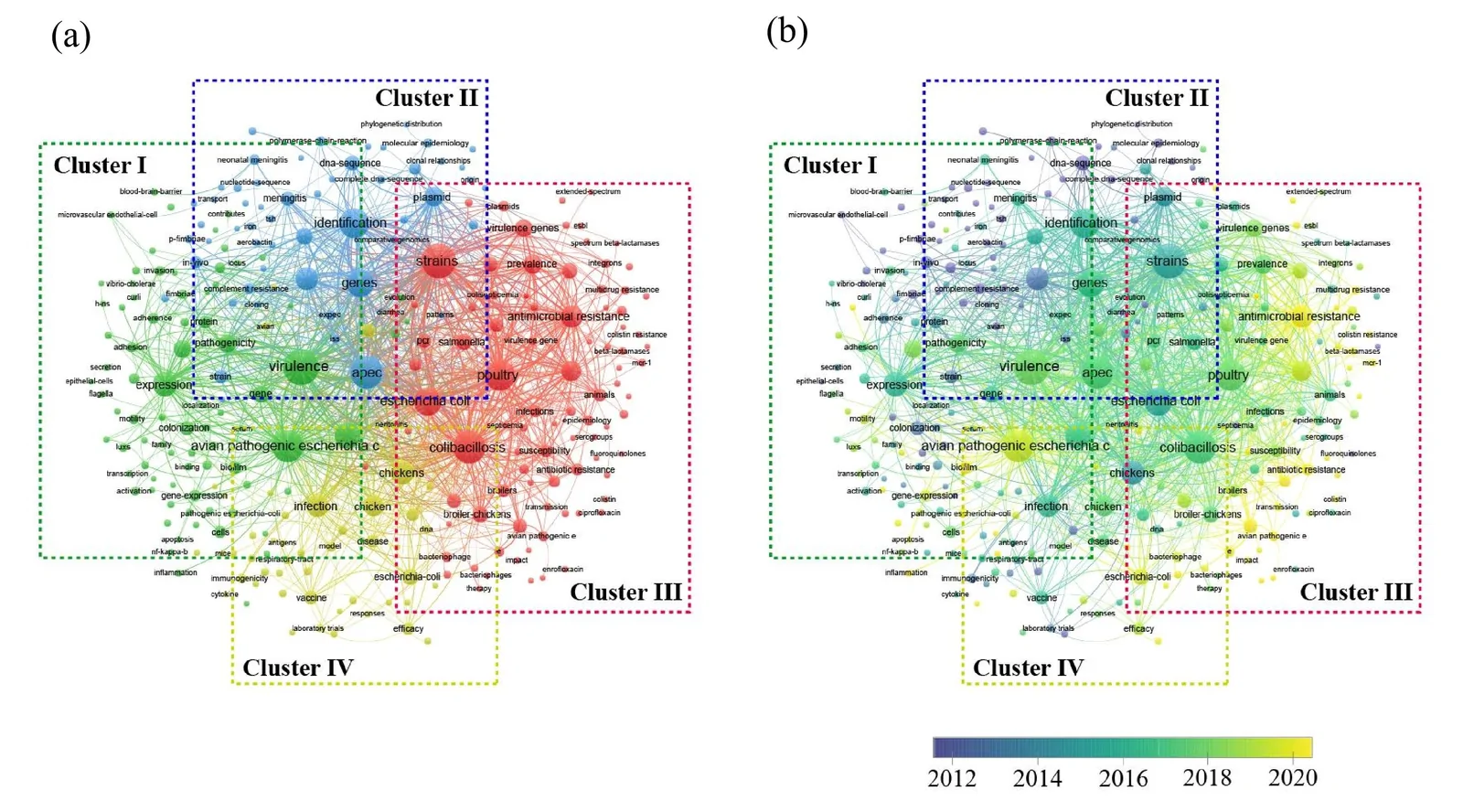
Keyword co-occurrence analysis of APEC research: (**a**) network visualization and (**b**) overlay visualization. Each node represents a keyword, and node size is proportional to its occurrence frequency. Links indicate the co-occurrence of two keywords within the same publication, while line thickness reflects the strength of their co-occurrence. In panel (**a**), node colors and dashed boxes distinguish keyword clusters comprising terms with a high degree of thematic similarity. In panel (**b**), the color gradient from blue to yellow represents the average year of keyword occurrence, with blue indicating earlier occurrences and yellow indicating more recent occurrences. The analysis was performed using VOSviewer, with a minimum keyword occurrence threshold of 10.

Keyword clustering analysis uses statistical algorithms to group keywords that frequently co-occur, thereby delineating the thematic structure and intellectual connections within a research field [34]. In the present study, the keyword co-occurrence network revealed four major thematic clusters (Figure 4).

Cluster I represents the principal research themes related to the pathogenic mechanisms of APEC. In the keyword co-occurrence network, “avian pathogenic *Escherichia coli*”, “virulence”, “pathogenicity”, “gene”, expression”, “colonization” occupy central positions. Among these, the relatively large nodes for “virulence” and “avian pathogenic *Escherichia coli*” are closely linked to “gene”, “expression”, “protein”, “biofilm”, and “cells”. These associations suggest that research in this cluster has primarily focused on virulence-related phenotypes and their molecular regulation, with increasing attention to bacterial colonization, tissue invasion, environmental adaptation, and host immune damage.

Keywords including “adhesion”, “adherence”, “binding”, “fimbriae”, “P fimbriae”, and “epithelial cells” are primarily associated with bacterial attachment to host cells during the early stages of infection. APEC adheres to respiratory epithelial cells, fibroblasts, and vascular endothelial cells through fimbriae, outer membrane proteins, and autotransporter adhesins, thereby promoting persistence on host tissue surfaces and subsequent colonization [35,36,37,38]. A newly identified group of P-like fimbriae, designated PL fimbriae, enhances bacterial adherence to bladder and kidney epithelial cells, promotes biofilm formation, and confers a competitive advantage during kidney colonization. These findings suggest that fimbrial structures contribute to host adaptation and tissue tropism in APEC and other ExPEC strains [36].

Keywords related to adhesion, including “fimbriae”, “flagella”, “motility”, and “secretion”, further suggest that APEC relies on a coordinated set of surface structures and associated regulatory systems to establish infection. These systems collectively mediate attachment, motility, and secretion, enabling APEC to overcome host barriers, establish contact with host cells, and disseminate through tissues. Inactivation of *ygeK* in the ETT2 region markedly reduces flagellar formation, motility, serum resistance, and cellular adhesion, ultimately attenuating virulence [39]. Similarly, deletion of *etrA* reduces the expression of fimbria-associated genes and impairs bacterial survival in macrophages and proliferation in ducks. Together, these findings suggest that regulatory factors associated with secretion systems can influence bacterial invasion and persistence by modulating the expression of surface structures [40].

The keywords “colonization” and “invasion” reflect, respectively, the ability of APEC to persist in the host and to cross tissue barriers. Their associations with “microvascular endothelial cell”, “blood-brain barrier”, “respiratory tract”, and “cells” suggest that research has extended beyond the initial respiratory infection to encompass bloodstream dissemination, colonization of visceral organs, and traversal of barriers within the central nervous system. Deletion of the *cpdB* gene slows bacterial growth, causes morphological changes, and markedly reduces the expression of *ompA*, *fur*, *ftsZ*, and *tnaA*. Consequently, the mutant strain exhibits a reduced capacity to persist in the lungs, liver, and spleen of chickens. These findings suggest that APEC colonization relies on the coordinated maintenance of multiple physiological processes, including cell division, membrane integrity, metabolic activity, and iron homeostasis [41].

During invasion and systemic dissemination, iron acquisition and serum resistance, represented by the keywords “aerobactin”, and “complement resistance genes” are important determinants of APEC pathogenicity. Given the limited availability of iron in the host, APEC relies on siderophore-mediated iron acquisition systems, including the aerobactin system, to support growth in the bloodstream and internal organs. The *iucB* gene is involved in aerobactin biosynthesis, whereas *iutA* encodes its cognate outer-membrane receptor. Disruption of either gene reduces bacterial loads in the heart, liver, kidneys, spleen, and lungs, while simultaneous deletion of both genes produces a more pronounced attenuation of virulence. These findings indicate that effective iron acquisition depends on the coordinated functions of siderophore biosynthesis and receptor-mediated uptake [42]. Complement resistance may further facilitate systemic infection by protecting APEC against serum-mediated bactericidal activity. Strains carrying the *iss* gene are more frequently associated with serum resistance and embryonic lethality [43].

The notable presence of keywords such as “expression”, “gene expression”, “transcription”, and “activation” in Cluster I indicates that APEC research addresses not only the identification of virulence genes but also the regulatory networks and environmental sensing mechanisms involved in their expression. For example, the CitA/CitB two-component system regulates genes involved in biofilm formation, membrane protein expression, and virulence, thereby linking metabolic sensing to colonization, antimicrobial resistance, and pathogenicity [44]. Alongside this focus on bacterial regulation, keywords such as “inflammation”, “cytokines”, “NF-κB”, and “apoptosis” indicate growing interest in host responses to APEC infection. In chicken macrophages, differentially expressed microRNAs have been implicated in the regulation of immune-related pathways, including MAPK, mTOR, TGF-β, and autophagy signalling. In particular, gga-miR-181b-5p targets TGFBR1 and modulates TGF-β signalling, highlighting the role of host post-transcriptional regulation in the response to APEC infection [45]. Taken together, the composition and interconnections of Cluster I reflect a broader shift in APEC pathogenesis research towards the regulatory processes that shape host–pathogen interactions.

Cluster II encompasses molecular detection, virulence gene profiling, plasmid architecture, and comparative genomics in APEC research. In the keyword co-occurrence network, “genes” is represented by the largest node and is closely associated with “DNA sequence”, “polymerase chain reaction”, “molecular epidemiology”, and “comparative genomics”. Earlier studies used PCR to screen APEC isolates for candidate virulence genes, including *iss*, *iucD*, *iutA*, *tsh*, *papC*, *irp2*, *vat*, *cva*, and *cvi*, and used the resulting profiles to assess the pathogenic potential. Among these genes, *iss* is highly conserved across strains from different serogroups and geographical regions and has therefore been investigated as a candidate marker for APEC detection [46]. TaqMan real-time PCR assays targeting *ompT*, *hlyF*, *iroN*, *iutA*, and *iss* were evaluated in singleplex and duplex formats and showed high concordance with conventional endpoint PCR [47]. Although different strains may share some virulence determinants, the pathogenic potential of the isolates can vary considerably [48]. Thus, no single gene can reliably distinguish APEC isolates from other avian *E. coli* strains, because APEC pathogenicity is a complex polygenic trait rather than one defined by a single conserved molecular marker [49].

The notable presence of the “plasmid” node highlights the diversity and modular organization of APEC virulence genes, many of which are carried on large ColV or ColBM plasmids and are associated with iron acquisition, serum resistance, adhesion, haemolysis, and bacteriocin production. Full-length sequencing and segmental deletion mutagenesis of the virulence plasmid pVM01 showed that its conserved region contributes to air-sac colonization and lesion development, while both conserved and variable regions contribute to tracheal colonization [50]. Keywords such as “complete DNA sequence” and “comparative genomics” point to the use of sequencing-based approaches to examine not only individual virulence plasmids but also the APEC plasmidome. Hybrid assembly that combines Nanopore long reads with Illumina short reads has revealed a more complex APEC plasmidome than can be resolved using short reads alone. In one study, Sanderson et al. identified 79 plasmids from 19 APEC isolates, illustrating how short-read data alone may underestimate plasmidome complexity and obscure the linkage between antimicrobial resistance and virulence determinants [51]. Certain conjugative plasmids harbor both antimicrobial resistance genes and APEC virulence determinants on the same plasmid backbone, implying that this genetic linkage may facilitate the co-transfer of antimicrobial resistance and virulence traits [52]. Keywords related to molecular epidemiology, clonal relationships, phylogenetic distribution, origin, and comparative genomics indicate a strong focus on the clonal dissemination of APEC. Whole-genome sequencing, multilocus sequence typing (MLST), and phylogenetic analysis have been used to elucidate clonal relationships and reconstruct transmission patterns by comparing core and accessory genomes, pathogenicity islands, and plasmid profiles. The genome of APEC strain O1:K1:H7 shows a high degree of homology with those of several human-derived ExPEC strains, and most of its genetic content is also found in human ExPEC isolates, suggesting that there is no clear genetic boundary between avian- and human-derived isolates [33]. The “neonatal meningitis” and “meningitis” nodes are located in the upper region of Cluster II and are associated with the “identification” node, highlighting research on the genetic relationships between APEC and ExPEC strains associated with human extraintestinal infections. This interpretation is supported by previous studies showing that avian O1:K1:H7 isolates share several determinants involved in iron acquisition and serum resistance with human-derived UPEC and NMEC strains. In addition, some phylogroup B2 O1:K1:H7 isolates recovered from poultry and humans cluster within the same clonal lineages. Together, these findings suggest that certain APEC lineages are not strictly host-specific and may have the potential to colonize humans and cause extraintestinal infections [53]. However, genomic similarity does not necessarily imply comparable pathogenic potential in a given host. Although APEC O1 belongs to ST95 and carries multiple virulence factors associated with neonatal meningitis, it produces lower bacteremia levels and less severe meningoencephalitic lesions in neonatal rats than prototypical human ExPEC strains [54]. Overall, Cluster II reflects a shift in the molecular epidemiological study of APEC from the identification of individual genetic markers to integrated analyses of virulence gene profiles, plasmid architecture, population evolution, and host adaptation.

Cluster III focuses primarily on the epidemiology and antimicrobial resistance of APEC. In the keyword co-occurrence network, “strains”, “poultry”, “antimicrobial resistance”, “prevalence”, and “colibacillosis” appear as relatively large nodes and are closely connected to “chickens”, “broilers”, “epidemiology”, “serogroups”, “susceptibility”, and “antibiotic resistance”. The co-occurrence of “prevalence”, “strains”, and “serogroups” reflects research on variation in the predominant serogroups, phylogenetic backgrounds, and resistance profiles of APEC across poultry populations, production systems, and geographic regions. For example, Thomrongsuwannakij et al. found that multidrug resistance was markedly more prevalent among APEC isolates from commercial broilers than among those from indigenous chickens, suggesting that differences in production practices and antimicrobial exposure may contribute to this disparity [55]. Keywords such as “colibacillosis”, “epidemiology”, and “clonal relationships” reflect a growing emphasis on tracing the transmission and clonal dissemination of outbreak-associated APEC strains within poultry populations, extending beyond the phenotypic characterization of individual isolates. Whole-genome sequencing studies have shown that APEC isolates recovered from different organs of the same affected chicken generally exhibit high sequence similarity, suggesting that sequencing selected representative isolates may help identify the predominant pathogenic clones circulating within a flock [56].

“Antimicrobial resistance”, “multidrug resistance”, and “antibiotic resistance” occupy central positions within this cluster, indicating that resistance surveillance has remained a major research theme. Long-term surveillance of APEC isolates from commercial broilers in South Africa showed that the overall prevalence of multidrug resistance exceeded 80% [57]. By contrast, a decade-long surveillance program in Portuguese broiler breeder flocks documented declining resistance to several antimicrobial agents, together with a lower prevalence of multidrug resistance over time [58]. Together, these studies illustrate the geographic and temporal heterogeneity of antimicrobial resistance among APEC isolates. The occurrence of keywords such as “extended-spectrum β-lactamases (ESBLs)”, “integrons”, and “antimicrobial resistance genes” further indicates that research has expanded beyond phenotypic resistance surveillance to include the genetic determinants and mobile elements involved in resistance dissemination. Studies conducted in Egypt and eastern China found a high prevalence of class 1 integrons among APEC isolates and frequent detection of resistance genes, including *tetA*, *tetB*, *bla*_TEM_, *sul1*, and *sul2* [59,60].

In the VOSviewer overlay map, nodes related to “antimicrobial resistance”, “prevalence”, “integrons”, “*mcr-1*”, and several antimicrobial agents are shown in yellow-green, suggesting that antimicrobial resistance epidemiology and resistance gene dissemination have remained active areas of research. Some APEC strains concurrently harbor mcr-1, extended-spectrum β-lactamase (ESBL)-encoding genes, and other clinically important resistance determinants. When carried on conjugative plasmids or other mobile genetic elements, these genes may be transferred horizontally between bacterial hosts, including across species. This genetic plasticity supports the potential role of APEC as a reservoir associated with the dissemination of multiple antimicrobial resistance genes [61]. The detection of multidrug-resistant APEC isolates and conjugative resistance plasmids in retail poultry meat, poultry production environments, and migratory wild birds further points to multiple potential dissemination routes involving the food chain, wildlife, and the wider environment [62,63]. In this context, the acquisition of clinically important resistance genes by virulence-associated APEC lineages, including ST95, could compromise treatment options in poultry and raise broader concerns for food safety and public health [64].

The co-occurrence of “bacteriophages” and “therapy” indicates growing interest in phage therapy within this cluster, particularly in response to the increasing threat of antimicrobial resistance. Lytic bacteriophages active against APEC have been isolated from poultry farm wastewater and slaughterhouse environments. In addition, appropriately designed phage cocktails have shown activity against multiple commonly encountered APEC serotypes, supporting the feasibility of developing preparations with broader strain coverage [65]. Preliminary animal studies suggest that aerosolized bacteriophages may provide effective prophylaxis against APEC infection by reducing mortality, whereas intramuscular administration has been reported to alleviate severe clinical signs of colibacillosis [66]. Together, these findings support the potential of phage therapy for both the prevention and treatment of APEC infection. Recent studies have further suggested selecting complementary phages according to the sequence types, virulence genotypes, and host ranges of target APEC isolates to broaden the lytic spectrum and reduce the emergence of phage-tolerant mutants [67]. Overall, antimicrobial resistance remains a major challenge in the prevention and treatment of APEC infections, underscoring the need for further investigation of effective antibiotic alternatives, particularly phage therapy.

Cluster IV encompasses APEC infection models, vaccine development, assessments of immune protection, and alternative strategies for disease prevention and control. In the keyword co-occurrence network, prominent terms such as “chicken”, “infection”, “vaccine”, “disease”, and “model” are closely connected to “antigens”, “immunogenicity”, “efficacy”, “laboratory trials”, “respiratory tract”, and “cytokines”. These associations indicate that the cluster is centered on experimental approaches to APEC infection and disease control in chickens and turkeys, including studies of bacterial pathogenicity, host immune responses, and intervention efficacy. Within this broader theme, the close interconnections among “model”, “infection”, and “disease” highlight the central role of experimental infection models in APEC research. Bacterial virulence, inoculation route, infectious dose, and host age can jointly affect lesion severity and contribute to differences in clinical manifestations across models. Embryo lethality, intravenous inoculation, and subcutaneous inoculation models have been used to distinguish highly virulent APEC strains from low-virulence strains. By contrast, results obtained with intratracheal inoculation models show limited concordance with those from other experimental systems, indicating that model selection should be guided by the infection stage and pathological features under investigation [68]. For example, models of salpingitis and yolk peritonitis in laying hens allow researchers to compare the colonization capacity and disease-associated pathology of different APEC strains in the oviduct, ovary, and abdominal cavity [69].

Within this vaccine-related theme, the prominence of “vaccine”, “antigens”, and “efficacy” reflects the diversification of APEC vaccine platforms beyond conventional inactivated bacterins, including live-attenuated and autogenous vaccines, mucosal delivery approaches, and recombinant vector platforms. An *aroA* mutant derived from a highly virulent APEC O78 strain showed a favorable safety profile in chicks and protected chickens and turkeys against lethal challenge with virulent APEC strains, supporting the use of metabolically attenuated strains as live-vaccine candidates [70]. Multivalent autogenous vaccines formulated against serotypes prevalent on individual farms reduced mortality and lesion scores in breeding hens and improved the growth performance of their broiler progeny [71]. The use of conserved adhesive structures, including type 1 and P fimbriae, as candidate antigens, together with attenuated *Salmonella* strains as delivery vectors, enhanced mucosal and humoral immune responses and showed the potential to provide cross-protection against multiple APEC serogroups [72]. Taken together, these findings suggest that future vaccine development should prioritize conserved antigens capable of inducing cross-strain protection and coordinated mucosal, humoral, and cellular immune responses, rather than focusing solely on serotype-specific antibody responses.

Nodal terms such as “responses”, “immunity”, and “cytokines” indicate that recent work has increasingly examined the immune mechanisms underlying APEC vaccine protection. Following stimulation with live, irradiated, or inactivated APEC, chicken peripheral blood mononuclear cells produced interferon-γ and interleukin-17A, with live and irradiated bacteria inducing significantly stronger responses than inactivated bacteria. These findings suggest that the form in which an antigen is presented can influence the magnitude and polarization of the cellular immune response [73]. Sadeyen et al. reported that both commercial live and inactivated vaccines induced APEC-specific IgY production and splenocyte recall responses. Notably, B-cell depletion almost completely abolished vaccine-mediated protection against homologous strains, indicating that humoral immunity is essential for homologous protection. However, the observed protection also depended on the combined contribution of humoral and cellular immune responses [74]. These findings indicate that vaccine efficacy cannot be evaluated adequately on the basis of serum antibody titers alone. A more comprehensive assessment should incorporate mucosal antibody levels, cytokine profiles, lymphocyte proliferation, and tissue bacterial loads.

#### 3.6.2. Keyword Burst Detection and Emerging Trends

**Figure 5:**
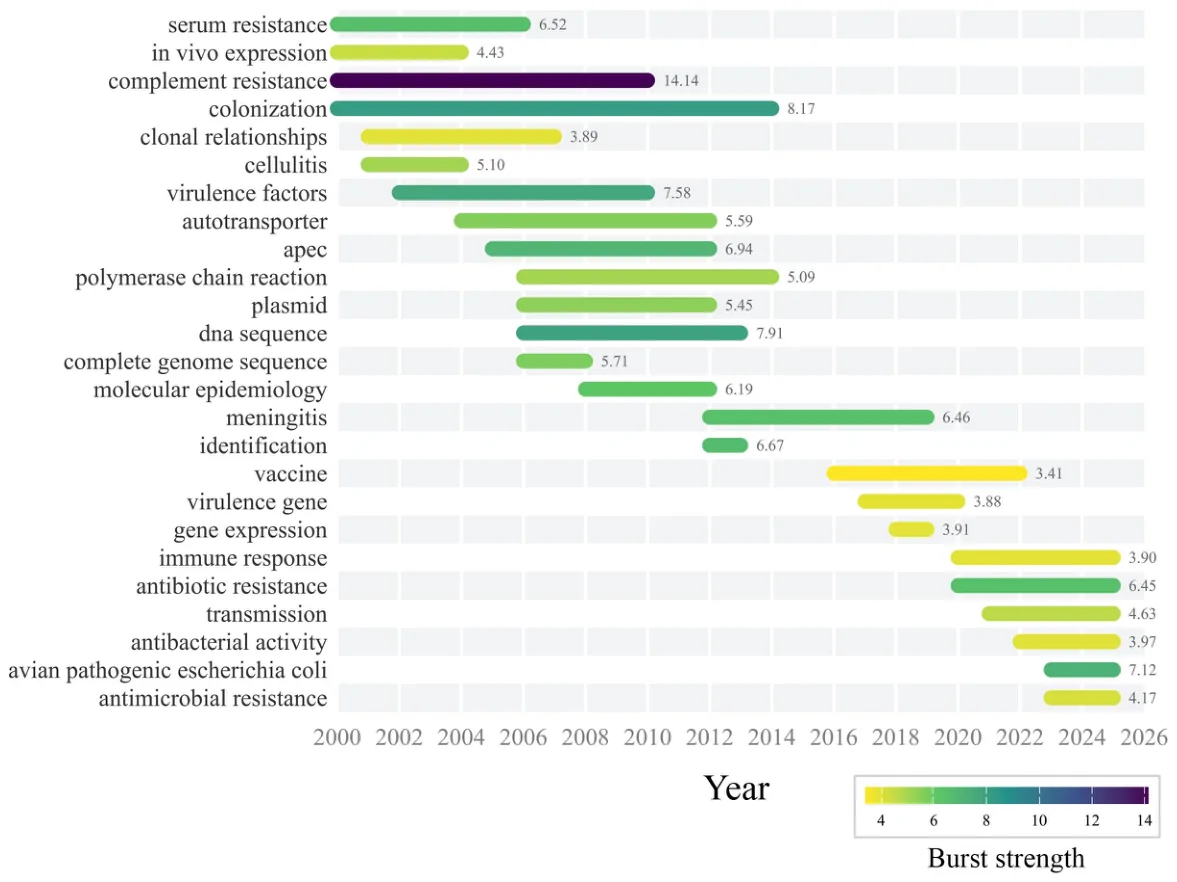
Keyword burst detection in APEC research.

Analysis of annual publication output and keyword burst strength indicates that APEC research evolved between 2000 and 2025 from an initial emphasis on pathogenic mechanisms to a broader research agenda encompassing molecular epidemiology, host immune responses, antimicrobial resistance dissemination, and associated public health risks. The field can be broadly divided into four phases: an initial phase (2000–2007), a growth phase (2008–2014), a period of accelerated development (2015–2019), and a mature phase (2020–2025) (Figure 5).

From 2000 to 2007, “complement resistance” showed the strongest keyword burst, with a burst strength of 14.14, indicating that early APEC research focused mainly on the mechanisms underlying bacterial survival, colonization, immune evasion, and virulence expression in the host. During the subsequent growth phase (2008–2014), research expanded beyond individual virulence factors and phenotypic traits to include molecular epidemiology, strain identification, sequence analysis, and the assessment of pathogenic risks across host specie. The genetic relatedness between APEC and human ExPEC also attracted increasing attention, bringing the potential zoonotic relevance of APEC and its implications for public health into greater focus.

During the accelerated development phase (2015–2019), research priorities broadened further to include vaccine development, functional characterization of virulence genes, transcriptional regulation, and the evaluation of disease prevention and control strategies. In the most recent phase (2020–2025), antimicrobial resistance and alternative antibacterial approaches became increasingly prominent, with antimicrobial resistance emerging as one of the major research themes in contemporary APEC studies. Overall, the temporal evolution of burst keywords broadly paralleled the increase in annual publication output and reflected the expanding scope of APEC research, from early investigations of pathogenic mechanisms and strain identification to more integrated studies of molecular epidemiology, genomics, vaccine-mediated prevention, antimicrobial resistance, and the implications of cross-host transmission.

### 3.7. Limitations

This study has several limitations. First, the analysis was based exclusively on the WoSCC. Although this database provides standardized, high-quality bibliographic records suitable for bibliometric analysis, relevant publications indexed only in other databases may have been omitted. Second, the inclusion of only English-language publications may have introduced language and geographical biases, resulting in the underrepresentation of studies published in other languages. Third, citation-based indicators are influenced by publication age, journal visibility, database coverage, and self-citation. Consequently, citation counts may not accurately reflect the scientific quality or practical significance of individual studies, and recent publications may be disadvantaged because they have had less time to accumulate citations.

## 4. Conclusions

In this study, we conducted a bibliometric analysis of the global literature on APEC published between 2000 and 2025, focusing on countries, research institutions, journals, cited references, and keywords. The results show that APEC research has developed from early studies of pathogen identification and virulence phenotypes into a multidisciplinary field involving molecular epidemiology, comparative genomics, antimicrobial resistance, host immunity, and disease prevention and control. The continued increase in annual publication output, particularly after 2019, suggests that APEC is receiving growing attention because of its effects on poultry health and production, as well as its possible implications for food safety, antimicrobial resistance, and public health.

China contributed the largest number of publications, whereas the USA and several European institutions had greater citation impact. This difference shows that publication volume does not necessarily reflect academic influence. It also highlights the importance of broader international collaboration, particularly in the sharing of isolates, genomic data, and epidemiological information. At the thematic level, virulence, colonization, invasion, plasmids, and bacterial strains remained central topics throughout the study period. However, research gradually moved beyond the characterization of individual virulence factors and increasingly addressed virulence-regulatory networks, mobile genetic elements, the co-transmission of antimicrobial resistance and virulence determinants, and transmission risks across hosts and production environments. The growing interest in vaccines, immune responses, bacteriophages, and other non-antibiotic interventions further suggests that the field is moving from describing pathogenic mechanisms toward developing practical approaches for disease prevention and control.

Future research should establish closer links between molecular findings, epidemiological evidence, and conditions in poultry production. Coordinated surveillance of APEC isolates from poultry, food, environmental sources, and human clinical samples should be promoted within a One Health framework. Whole-genome sequencing, combined with transcriptomic, proteomic, and metabolomic analyses where appropriate, may help clarify the genetic basis of virulence, antimicrobial resistance, persistence, and transmission. Standardized procedures for sampling, serotyping, genotyping, antimicrobial susceptibility testing, and risk assessment are also needed to make results more comparable across regions and studies. Particular attention should be paid to high-risk clonal lineages and their associated resistance plasmids, especially where they are repeatedly detected in different sources or host populations.

The practical value of candidate interventions also requires further assessment. Conserved antigens, anti-virulence targets, bacteriophage cocktails, and microecological preparations should first be tested in standardized animal challenge models and then evaluated in well-designed field studies. These studies should assess not only efficacy, but also strain coverage, duration of protection, safety, stability, and suitability for commercial poultry production. Overall, future progress in APEC research will depend on connecting genomic surveillance and mechanistic studies with evidence from field conditions. Such an approach may support more reliable strategies for reducing APEC-associated disease, limiting the spread of antimicrobial resistance, and addressing risks at the animal–food–environment–human interface.

## Figures and Tables

**Table 1:** Top 10 countries/regions by number of publications on APEC, 2000–2025.

Country	Records	%	SCP ^1^	MCP ^2^	MCP_Ratio	Total Cited Citations	Average Cited Citations
China	328	29.26	298	30	9.15	4592	14.00
USA	150	13.38	126	24	16.00	7332	48.88
Brazil	89	7.94	73	16	17.98	1640	18.43
Canada	41	3.66	31	10	24.39	1996	48.68
Iran	35	3.12	32	3	8.57	340	9.71
Egypt	33	2.94	19	14	42.42	646	19.58
France	32	2.85	21	11	34.38	1802	56.31
UK	32	2.85	21	11	34.38	1505	47.03
South Korea	28	2.50	22	6	21.43	453	16.18
Denmark	25	2.23	15	10	40.00	616	24.64

^1^ single-country publications. ^2^ multiple-country publications.

**Table 2:** The 10 most productive institutions in APEC research, 2000–2025.

Institution	Total Publications		Location	Total Citations	Average Citation per Paper
	No.	%			
Iowa State University	75	6.69	USA	4706	62.75
Yangzhou University	69	6.16	China	886	12.84
Anhui Agricultural University	65	5.80	China	839	12.91
Nanjing Agricultural University	65	5.80	China	1187	18.26
Chinese Academy of Agricultural Sciences	39	3.48	China	821	21.05
University of Copenhagen	30	2.68	Denmark	727	24.23
National Research Institute for Agriculture, Food and Environment (INRAE)	26	2.32	France	2060	79.23
University of Georgia	26	2.32	USA	855	32.88
North Dakota State University	25	2.23	USA	2952	118.08
University of Minnesota	25	2.23	USA	2224	62.75

**Table 3:** The 10 most productive journals in APEC research, 2000–2025.

Journal	Total Publications		IF_2025_	Total Citations	Average Citation per Paper
	No.	%			
*Poultry Science*	101	9.01	4.5	1481	14.66
*Veterinary Microbiology*	81	7.23	2.8	2549	31.47
*Avian Diseases*	60	5.35	1.3	1680	28.00
*Avian Pathology*	53	4.73	2.7	1359	25.64
*PLoS One*	39	3.48	2.8	1466	37.59
*Microbial Pathogenesis*	28	2.50	3.9	607	21.68
*Infection and Immunity*	28	2.50	3.4	2241	80.04
*Frontiers in Veterinary Science*	22	1.96	3.1	245	11.14
*BMC Veterinary Research*	20	1.78	3.1	505	25.25
*Microorganisms*	20	1.78	4.7	381	19.05

**Table 4:** Top 10 most cited publications on APEC, 2000–2025.

Title	First Authors	Year	Journal	Total Citation
Virulence factors, prevalence and potential transmission of extraintestinal pathogenic *Escherichia coli* isolated from different sources: recent reports	Sarowska, J	2019	*Gut Pathogens*	510
Avian pathogenic, uropathogenic, and newborn meningitis-causing *Escherichia coli*: How closely related are they?	Ewers C	2007	*International Journal of Medical Microbiology*	446
Identification of minimal predictors of avian pathogenic *Escherichia coli* virulence for use as a rapid diagnostic tool	Johnson, TJ	2008	Journal of Clinical Microbiology	370
Comparison of *Escherichia coli* isolates implicated in human urinary tract infection and avian colibacillosis	Rodriguez-Siek, KE	2005	Microbiology-sgm	354
The genome sequence of avian pathogenic *Escherichia coli* strain O1:K1:H7 shares strong similarities with human extraintestinal pathogenic *E. coli* genomes	Johnson, TJ	2007	Journal of Bacteriology	328
Characterizing the APEC pathotype	Rodriguez-Siek, KE	2005	Veterinary Research	312
Colibacillosis in poultry: unravelling the molecular basis of virulence of avian pathogenic *Escherichia coli* in their natural hosts	Dziva, F	2008	Avian Pathology	305
Avian pathogenic *Escherichia coli* (APEC): An overview of virulence and pathogenesis factors, zoonotic potential, and control strategies	Kathayat, D	2021	Pathogens	292
DNA sequence of a ColV plasmid and prevalence of selected plasmid-encoded virulence genes among avian *Escherichia coli* strains	Johnson, TJ	2006	Journal of Bacteriology	279
Comparison of extraintestinal pathogenic *Escherichia coli* strains from human and avian sources reveals a mixed subset representing potential zoonotic pathogens	Johnson, TJ	2008	Applied and Environmental Microbiology	226

## Data Availability

No new data were created or analyzed in this study. Data sharing is not applicable to this article.

## Supplementary Materials

The following supporting information can be downloaded at: https://www.mdpi.com/article/doi/s1.
